# Bioassembly of Myoblast Spheroids in Electrofibrillated Scaffolds for 3D Muscle Tissue Biofabrication

**DOI:** 10.1002/smll.202503351

**Published:** 2025-08-28

**Authors:** Corinna Heinze, Camilla Mussoni, Matthias Ryma, Csaba Gergely, Zan Lamberger, Anna Schäfer, Kristina Andelovic, Philipp Stahlhut, Gregor Lang, Jürgen Groll, Taufiq Ahmad

**Affiliations:** ^1^ Department of Functional Materials in Medicine and Dentistry Institute of Functional Materials and Biofabrication (IFB) and Bavarian Polymer Institute (BPI) Julius‐Maximilians‐Universität Würzburg 97070 Würzburg Germany; ^2^ Chair of Biomaterials Engineering Faculty, Prof.‐Rüdiger‐Bormann‐Straße 1 University of Bayreuth 95447 Bayreuth Germany

**Keywords:** extracellular matrix, melt electrofibrillation (MEF), melt electrowriting (MEW), muscle tissue engineering, spheroids

## Abstract

One of the key challenges in tissue engineering is recreating the extracellular matrix (ECM), which is essential for cell function, especially in anisotropic tissues like muscle, where tissue morphology dictates contraction and motion. The recently developed method of melt electrofibrillation offers a promising platform for producing highly aligned nanofibrillar scaffolds that mimic collagen. These scaffolds are created by melt electrowriting a blend of polycaprolactone (PCL) and polyvinyl acetate (PVAc), which are precisely printed in a box geometry. After washing out the PVAc, aligned nano‐scaled PCL fibrils remain. These fibers provide extracellular matrix‐like cues for cells to mature, encouraging them to align and interact with the structure. This study showcases the application of C2C12 muscle cells, which are assembled into spheroids and seeded on the scaffolds. Over 21 days, the cells align along the fibrils, colonize the scaffold, and, when exposed to differentiation media, begin forming early‐stage myotubes and express myogenic factors, such as Myogenin. This demonstrates that the synthetic matrix is a useful tool for studying the interaction of aligned 3D matrices in muscle tissue differentiation.

## Graphical abstract

This study explores the fabrication of nanofibrillar scaffolds through the melt electrofibrillation of polycaprolactone (PCL) to guide myogenic differentiation. The scaffolds provide nanofibrillar cues that promote cell organization and integration, mimicking key features of the extracellular matrix (ECM). The findings contribute to the development of bio‐inspired, sustainable scaffolds for tissue engineering and regenerative medicine applications.

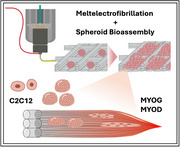

## 1 Introduction

The extracellular matrix (ECM) is a fundamental component of tissue architecture, providing not only structural support but also crucial biochemical and mechanical cues that regulate cellular behavior, including adhesion, migration, proliferation, and differentiation. In tissue engineering, replicating the intricate composition and architecture of the ECM is vital to designing scaffolds that can guide cell fate and foster the development of functional tissues.^[^ ^1^ ^]^ Collagen, the most abundant protein in the human body, is particularly important in this context, as it accounts for ≈90% of ECM proteins, especially in fibril‐forming tissues such as skin, bone, tendon, and muscle. Collagen type I forms hierarchical fibrillar structures that impart tensile strength and structural integrity, making it an ideal model for scaffold design.^[^ ^2^ ^]^

In natural tissues, the ECM's fibrous architecture, or nanotopography, plays a key role in directing cell behavior by providing topographical cues that influence cellular alignment and tissue organization.^[^ ^3^ ^]^ This is particularly evident in musculoskeletal tissues like tendons and muscles, where the highly aligned fibrillar bundles of collagen guide the organization of muscle fibers and enable the tissue to withstand mechanical stresses.^[^ ^4^ ^]^ Therefore, replicating the ECM's fibrous architecture is a critical challenge in tissue engineering, especially for applications aimed at regenerating mechanically active tissues such as muscle.^[^ ^5^ ^]^

A variety of scaffold fabrication techniques have been developed to mimic the complex structure of the ECM. One of the most widely used methods is electrospinning, which produces nanofibers that can closely resemble the fine structure of natural ECM.^[^ ^6^ ^]^ Electrospun scaffolds offer excellent control over fiber diameter, alignment, and porosity, which are key parameters for replicating the fibrous architecture of tissues. In particular, aligned electrospun fibers have shown great potential in muscle tissue engineering, where they promote the directional growth and differentiation of muscle cells (myoblasts), facilitating the formation of functional muscle fibers.^[^ ^7^ ^]^

While electrospinning has proven effective for creating ECM‐like structures, it has limitations in terms of reproducibility and precise control over the hierarchical organization of fibers.^[^ ^8^ ^]^ Emerging technologies like three‐dimensional (3D) printing and melt electrowriting (MEW) offer more sophisticated means of fabricating ECM‐inspired scaffolds with enhanced structural control.^[^ ^9^ ^]^ MEW, in particular, allows for the precise deposition of micro‐ and nanofibers in well‐defined patterns,^[^ ^10^ ^]^ enabling the creation of fibrillar bundles that mimic the hierarchical arrangements of collagen in native tissues.^[^ ^11^ ^]^ This technology leverages electrostatic forces to produce fibers with controlled alignment and bundling, making it an excellent platform for replicating the complex architecture of tissues such as muscle and tendon.^[^ ^12^ ^]^

However, scaffold fabrication alone may not be sufficient to replicate the complexity of natural tissues fully. To create truly functional 3D tissues, it is an advantage to combine scaffold technologies with cellular building blocks that can organize into higher‐order structures.^[^ ^13^ ^]^ In recent years, 3D cell aggregates, such as spheroids, have emerged as valuable tools for tissue engineering.^[^ ^14^ ^]^ Spheroids, which consist of densely packed cell clusters, can already mimic the 3D cell‐cell interactions and microenvironment found in the early developmental stages of tissue formation. When used as building blocks for biofabrication, spheroids can self‐assemble into larger, more complex tissue constructs, offering a promising approach for generating functional, tissue‐like structures.^[^ ^15^ ^]^ Integrating ECM‐inspired scaffolds with cell‐based bioassembly techniques, such as spheroid fusion, presents a powerful strategy for engineering 3D tissues.^[^ ^16^ ^]^ By combining the structural guidance provided by aligned fibers with the biological complexity offered by spheroids, researchers can assemble tissue constructs that more closely resemble the native architecture and function of real tissues.^[^ ^17^ ^]^ This approach not only improves the structural and mechanical properties of engineered tissues but also enhances cell viability, differentiation, and overall functionality.

In this study, we propose the use of collagen‐mimicking melt‐electro‐fibrillated (MEF) scaffolds for generating an aligned nanofibrous matrix^[^ ^18^ ^]^ to address a major limitation of traditional muscle tissue engineering platforms that rely on 2D aligned fibers: the inability to support 3D muscle‐like structure formation. Our approach utilizes melt electrofibrillated rectangular microbundles to guide spheroids into aligned muscle tissue architectures spatially. This design recapitulates both the micro‐ and nano‐scale hierarchy of native muscle ECM in vitro. We want to use this synthetic material to provide microenvironmental matr cues to promote muscle cell maturation and differentiation. A high concentration of cells on the scaffold interacting with the fibrils is achieved through the bioassembly of spheroids. In particular, as tissue of interest for the case, we target the muscle, a very organized tissue where cell alignment is a fundamental aspect that guides myogenic differentiation.

## 2 Results

**Figure 1:**
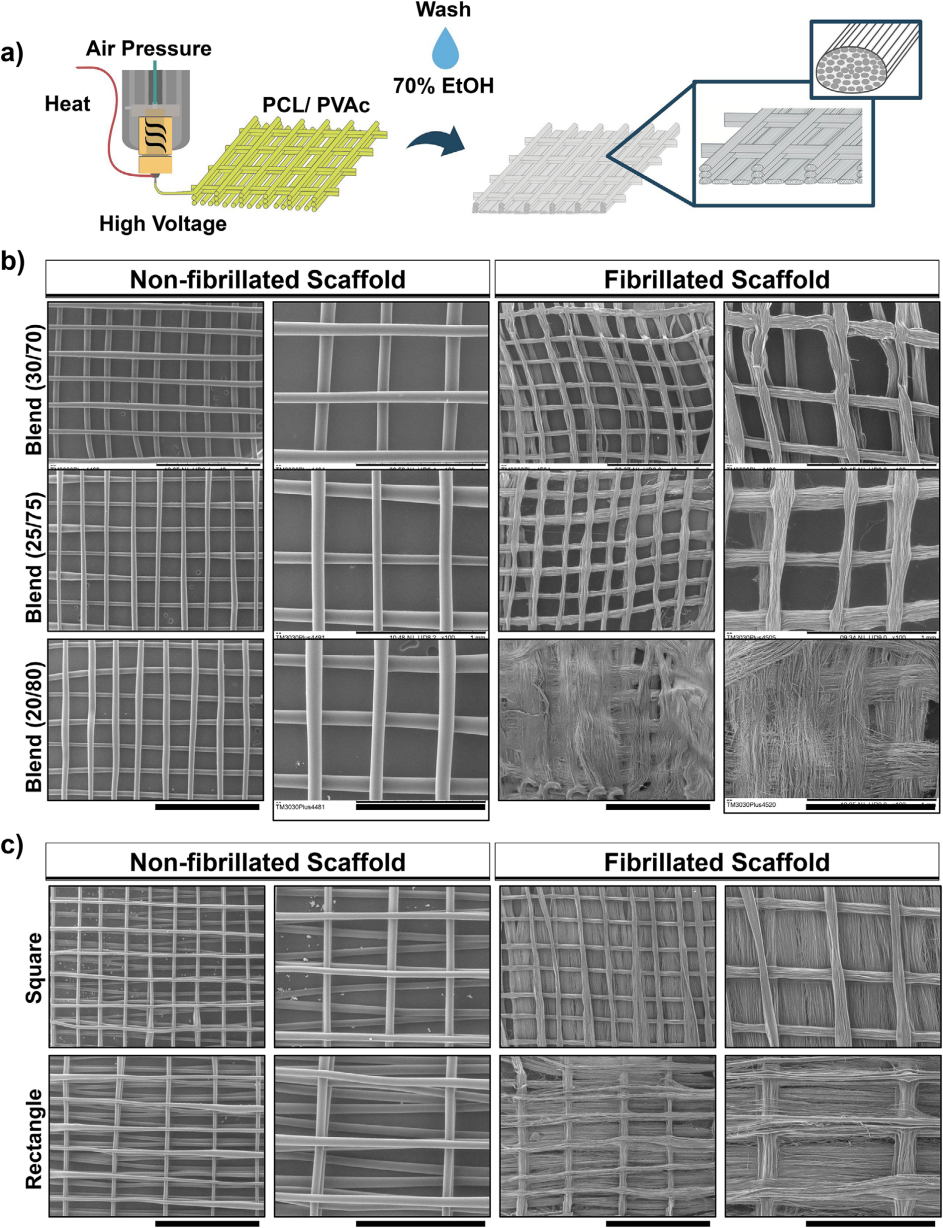
Melt electrofibrillated scaffolds: a) Graphic schematic of the electrofibrillation process, from meltelectrowriting of the scaffolds to fibrillation occurring after washing in ethanol. b) prints in squared boxes of different blends of PCL/PVAc fiber and consequent resulting fibril structures. Scale bar: 2 mm, Left and 1 mm, right. c) Scaffold printed with the internal geometry of square or rectangular boxes on the left and the resulting fibrillated scaffolds on the left. Scale bar: 2 mm, Left and 1 mm, right.

The nanofibrillated scaffolds were fabricated using a melt electrofibrillation method, where PCL‐PVAc polymer blend fibers were printed to form the scaffold. After washing, the fibers disbanded into smaller, nano‐sized fibers, as shown in **Figure** **1**a. The degree of fiber disintegration varied with the different polymer blends. Blends with a higher PCL content (70/30) resulted in more stable and compact fibril bundles following PVAc dissolution, while blends with a lower PCL content (80/20) were more unstable, making the scaffolds difficult to handle and less able to maintain their shape fidelity (Figure 1b). The fibrillated scaffolds (30/70 blend) exhibited stable microbundle formation, with both square and rectangular box structures (Figure 1c; Figure S1a,b, Supporting Information). The box dimensions of the scaffold before washing were 0.49 ± 0.3 mm by 0.48 ± 0.4 mm for square shapes and 0.94 ± 0.7 mm by 0.50 ± 0.6 mm for rectangular shapes. After washing, the square boxes measured 0.46 ± 0.6 mm by 0.48 ± 0.4 mm, while the rectangular boxes measured 0.98 ± 0.7 mm by 0.47 ± 0.5 mm (Figure 1c; Figure S1b,c, Supporting Information). Notably, we observed that rectangular‐shaped scaffolds exhibited a more stable form for handling the multi‐step processing. At the same time, the anisotropic and elongated structure is a better‐suited geometry for muscle engineering, and the added support by the catching fibers further stabilizes the nanofibril bundles. Therefore, we selected the scaffolds with the rectangular box shape for further experiments. A more detailed image analysis revealed the distribution of nanofiber diameter, having an average fiber size of ≈350 nm (Figure S1d, Supporting Information).

**Figure 2:**
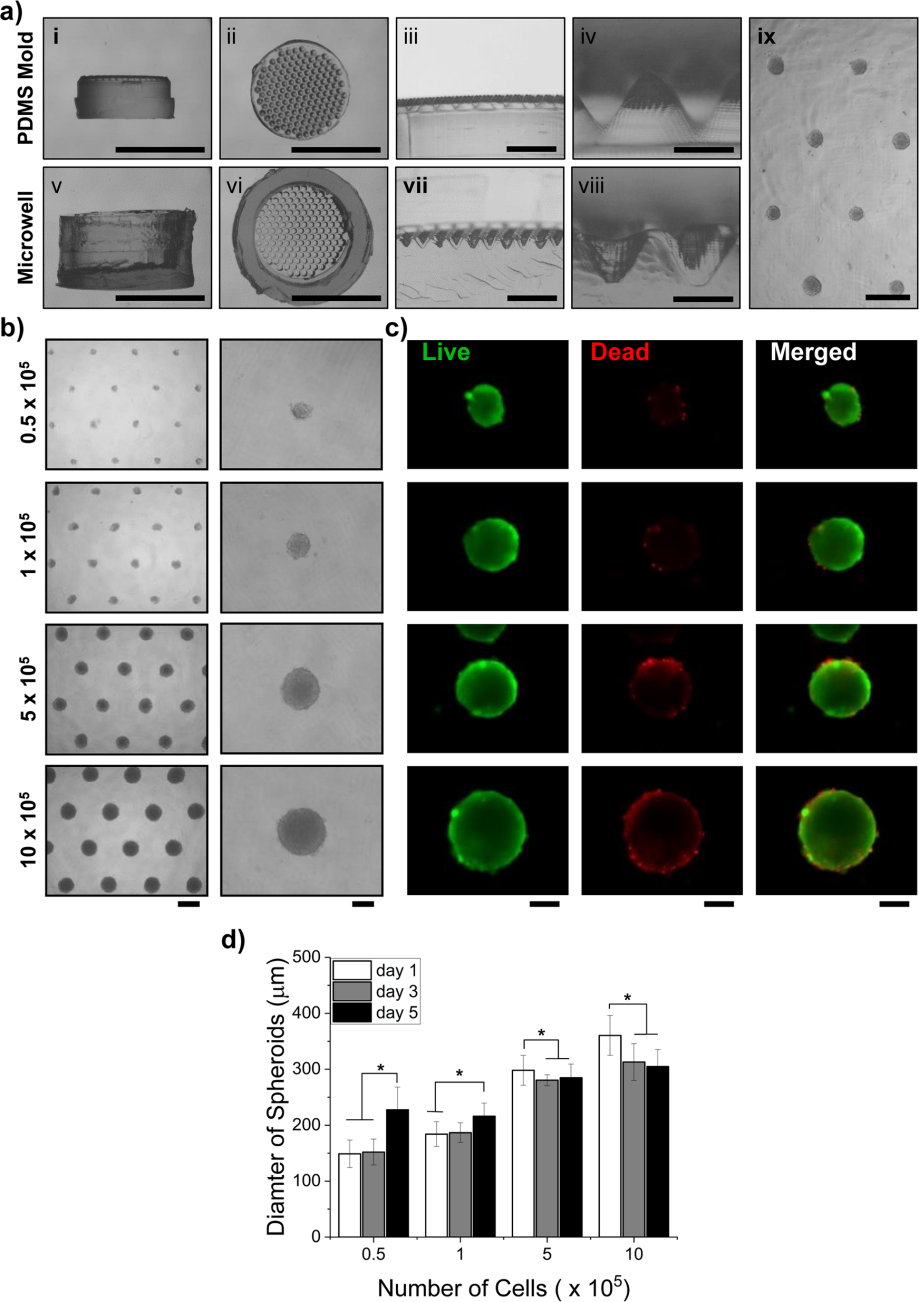
Spheroid fabrication: a, i‐iv) PDMS micropillar template at different magnifications; a, v‐viii) Agarose microwells derived from casting on the negative template; a, ix) resulting microwells visualized on optical microscope. Scale bar: i, ii, v, vi 10 mm, iii, vii 2 mm, iv, viii, ix 500 µm. b) spheroid in the wells with different numbers of cells. Scale bar: 500 µm Left, 200 µm Right. c) Live‐dead imaging of the spheroids of different sizes performed on day 5. Scale bar: 200 µm. d) Spheroid diameter evaluation.

The C2C12 cells seeded in honeycomb‐inspired cell‐clustering microwells formed spheroids after 24 h (**Figure** **2**a). The microwells were fabricated by casting agarose on PDMS micropillar stamps to generate microwells of 500 µm. Initially, the microwells had a hexagonal shape, but they gradually transitioned to a round shape at the bottom, allowing for cell clustering. In addition to enabling the scale‐up production of spheroids, the honeycomb‐inspired microwells offer control over spheroid size by simply adjusting the cell number (Figure 2b,c). The diameter of the spheroids increased with the cell number, indicating a significant difference. We also observed an increase in the number of dead cell signals with a higher number of cells per spheroid after 24 h (Figure 2c). This trend was consistent on days 3 and 5, as observed in the live/dead staining of spheroids (Figure S2a,b, Supporting Information). The spheroid diameter after 24 h showed an increasing trend with 0.5 x 10^5^ and 1 x 10^5^ cells per spheroid (Figure 2d). However, spheroids with a large number of cells exhibited a shrinkage in diameter on days 3 and 5, while spheroids with fewer cells showed a significant increase in diameter by day 5.

**Figure 3:**
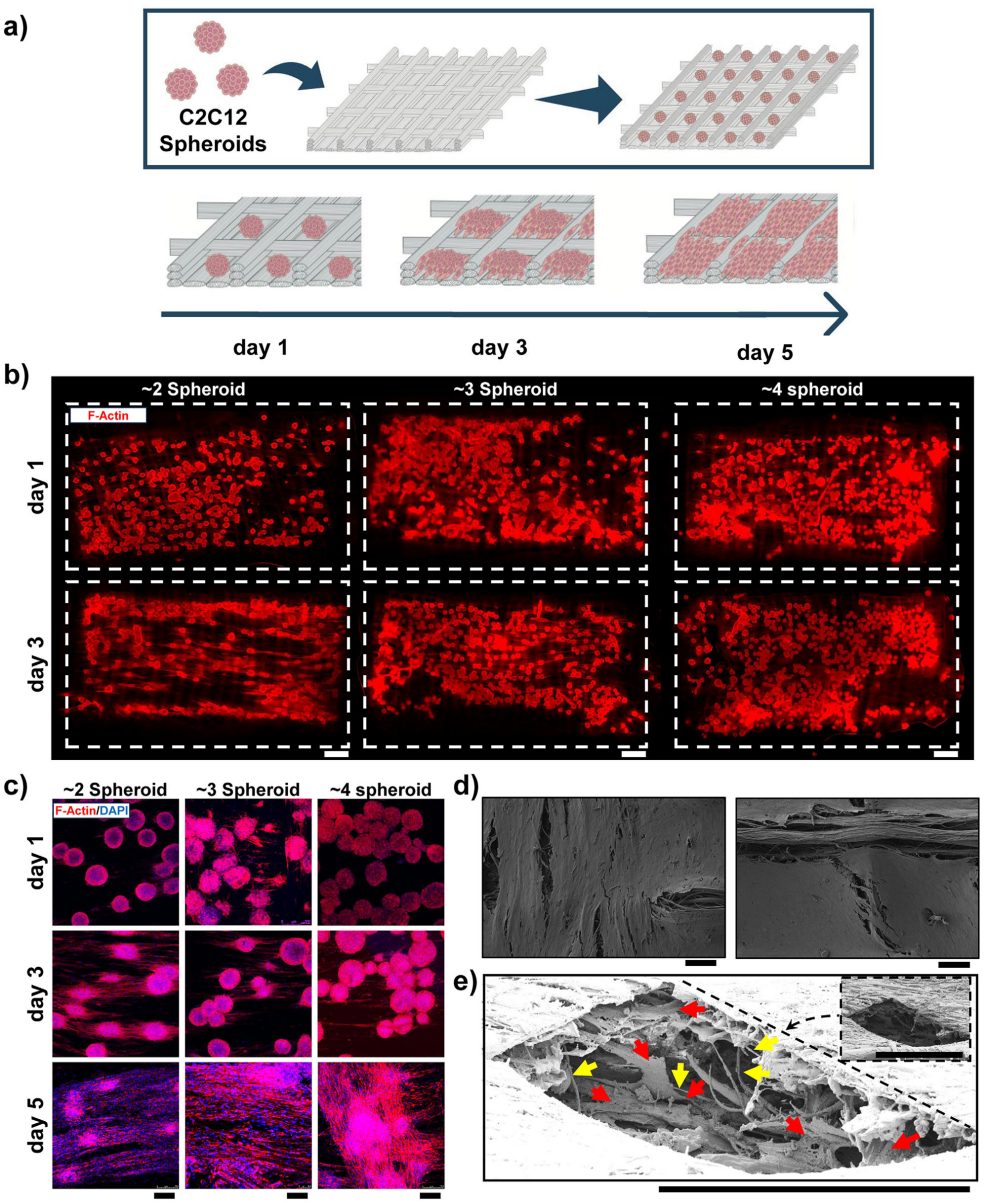
Scaffold seeding a) schematic representation of scaffold seeding of the spheroids inside the boxes and following maturation. b) F‐actin staining of different number of spheroids in the boxes over 3 days. Scale bar: 500 µm. c) F‐Actin and DAPI staining of spheroids in fibrillated boxes over 5 days. Scale bar: 250 µm. d) SEM imaging of cell distribution on the fibrils. Scale bar: 100 µm. e) focused ion beam SEM imaging of scaffold cell infiltration in 3 dimensions. Scale bar: 10 µm.

Next, the number of spheroids per chamber was screened to optimize cell density for effective scaffold coverage. On day 3, the spheroids were seeded onto the scaffold assembled into in‐house‐designed and 3D printed inserts (Figure S3a, Supporting Information). Different numbers of spheroids were seeded into each scaffold box (**Figure** **3**a) by pipetting the number of spheroids per box times the number of boxes on top of the scaffold and letting the spheroids homogeneously distribute in the boxes.T he cellularized constructs were then cultured for an additional 5 days (Figure 3a). F‐actin staining revealed that cells began migrating from the spheroids and covering the scaffold, a process that was most pronounced on day 3 (Figure 3b). The most homogeneous results were obtained with the boxes containing four spheroids. By day 5, cell coverage was significantly more pronounced (Figure 3c), and this was further confirmed by SEM imaging, which showed that the spheroids had filled the scaffold boxes with cells (Figure 3d). To further examine scaffold coverage, SEM imaging on day 5 revealed the formation of uniform cell layers on the scaffolds (Figure 3d), and FIB‐SEM analysis confirmed the presence of cells within the nanofibrillar bundles of the scaffold (Figure 3e). As control, PCL unfibrillated scaffolds alone exhibited sparse cell coverage, with spheroids or individual cells both clumping to form aggregates on the surface prone to detaching (Figure S3b,c, Supporting Information).

**Figure 4:**
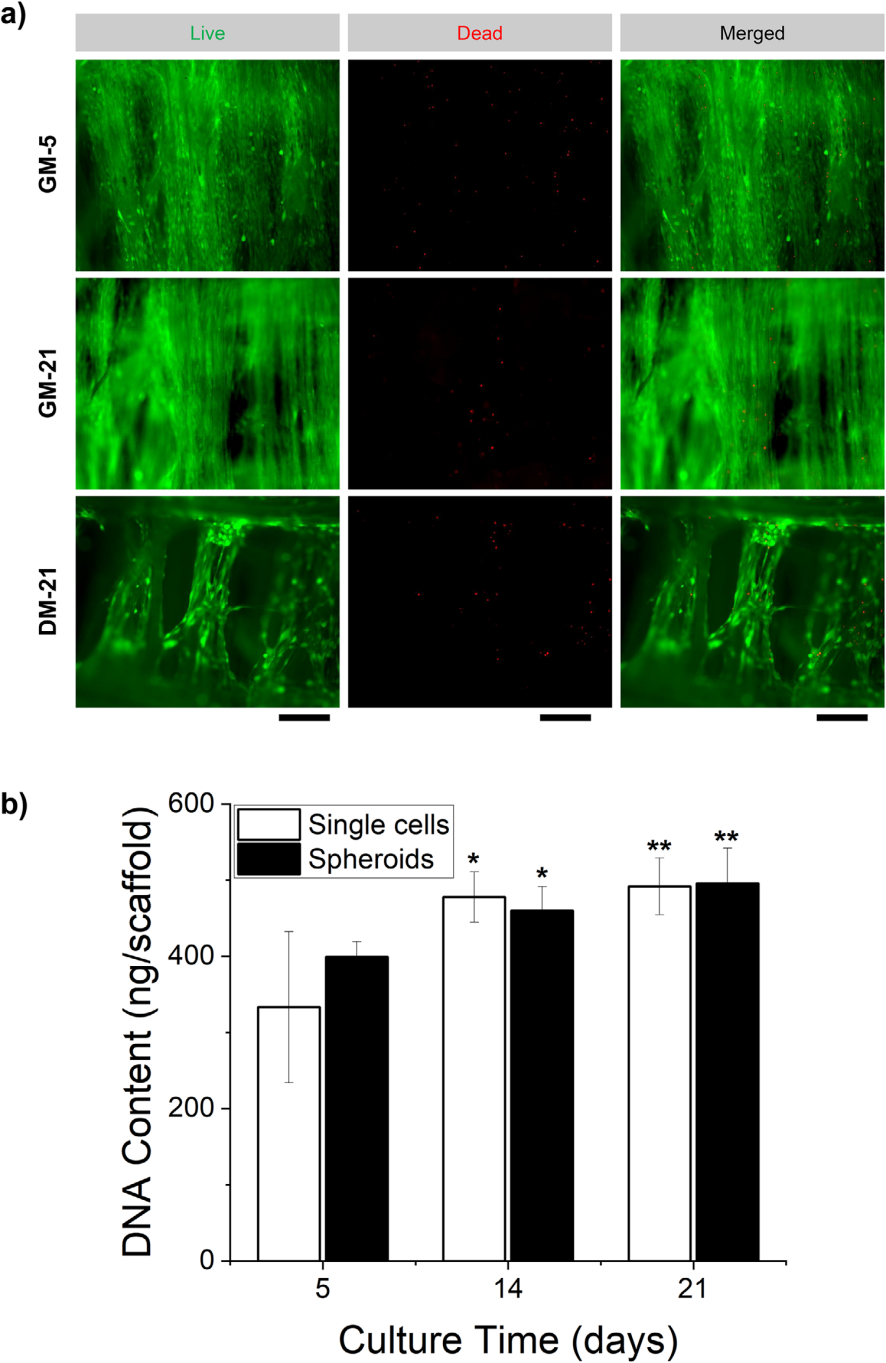
Long term culture on the scaffold: a) live and dead imaging of cell cultured under growth media (GM) and differentiation media (DM) from 5 to 21 days. Scale bar: 200 µm. b) DNA content evaluation between single cells and spheroids over 21 days.

The so‐described assemblies have been further cultivated for 21 days under two different media conditions, with growth media and with differentiation media, to highlight the effect of the fibrillar structures on maturation. From the live‐dead staining performed at day 5 and 21, we can show the high viability of the cells on the scaffold and how well distributed they are on the fibrils, with better coverage for the growth media compared to the differentiation media (**Figure** **4**a). Subsequently, we compared the DNA content of the same number of cells seeded on the scaffolds with either single cells or spheroids to evaluate proliferation. From the tests, we can see an increase in the amount of DNA over a longer time in culture (Figure 4b) compared to only seeding single cells.

**Figure 5:**
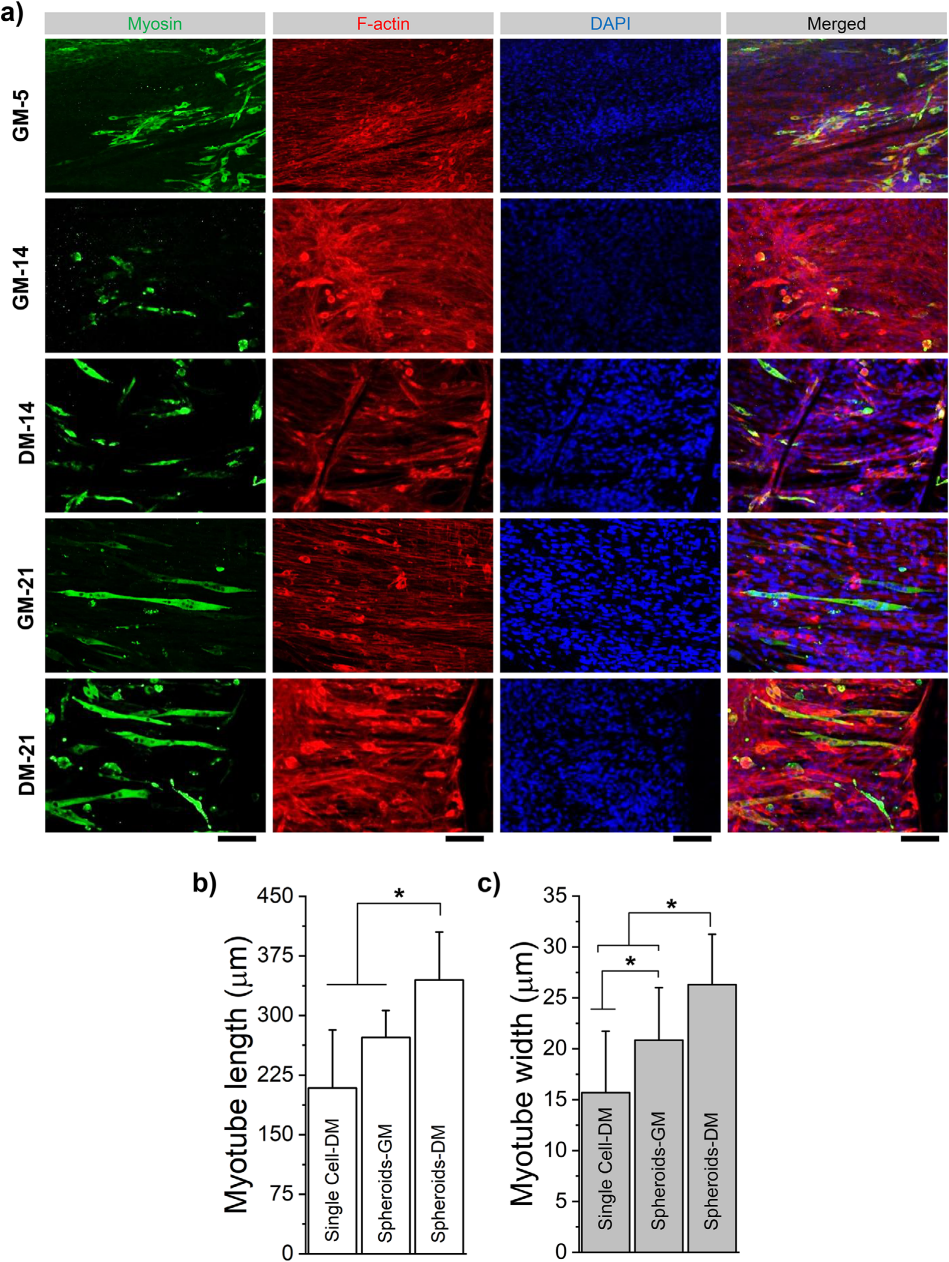
Immunochemistry a) myosin heavy chain staining using the MF20 antibody, DAPI and F‐Actin staining for cells over 21 days under growth media (GM), and differentiation media (DM) media conditions. Scale bar: 500 µm. b,c) Myotube formation is analyzed by quantification of length and width.

To prove myogenic activity from the C2C12 cells, we stained for myosin as well as F‐actin and DAPI (**Figure** **5**a; Figure S4a, Supporting Information). We confirm cell elongation and alignment along the fibers and the formation of myotubes as longitudinal bundles of cells. We evaluated their length and width for different seeding styles and media conditions (Figure 5b,c), and the images showed that signficantly longer and wider myotubes are formed on the fibrils seeded with spheroids in differentiation media.

**Figure 6:**
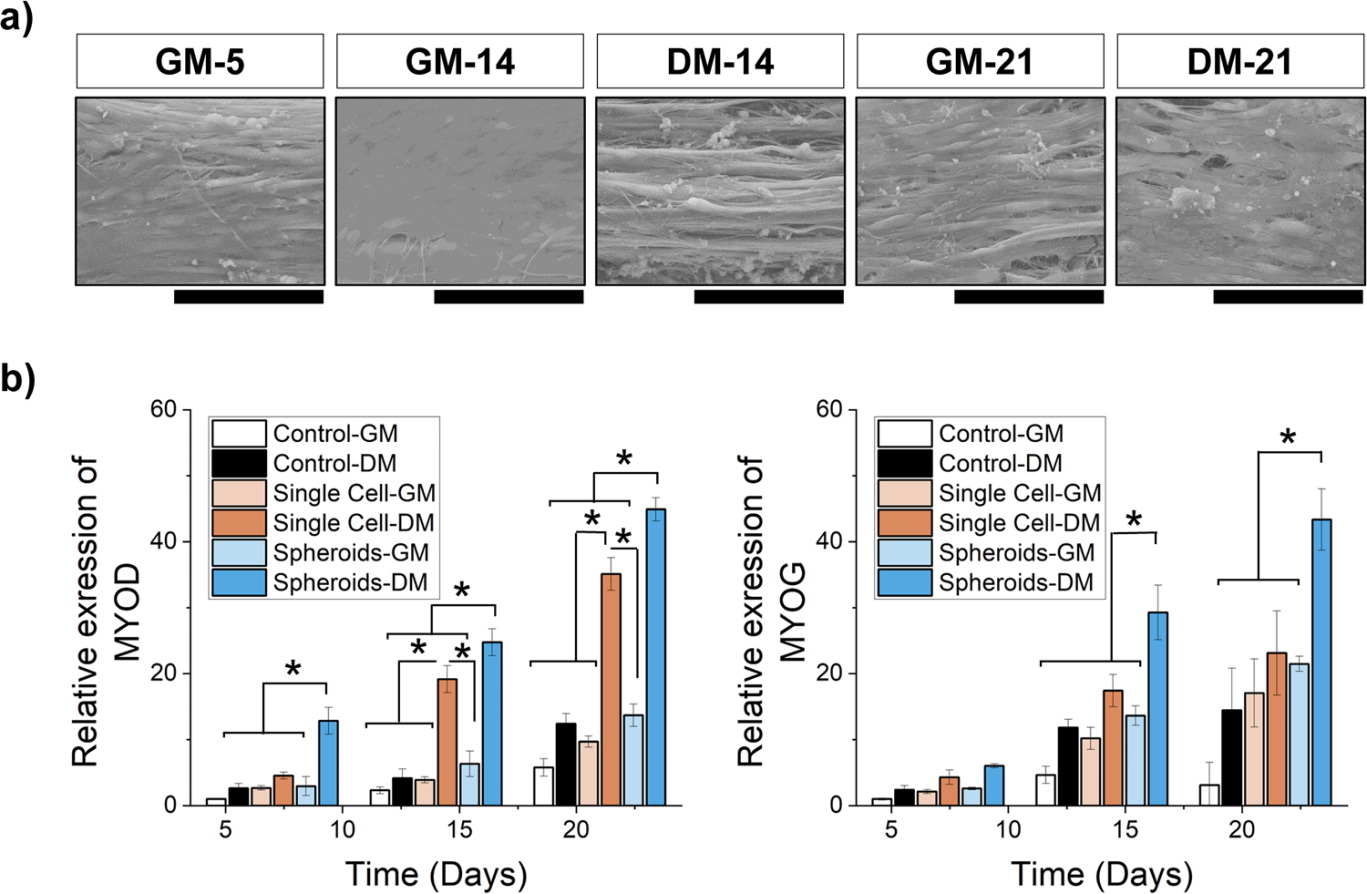
a) SEM imaging of cells on the fibrillated scaffold after 35 days under growth media (GM) or differentiation media (DM). b) Relative expression of MYOD and MYOG in cells cultivated in 2D (Control) or on fibrillated scaffolds, either as single cells or spheroids. Data are shown over 1–21 days for GM (lighter shade) and DM (darker shade) conditions.

A further analysis through SEM images shows how cells fully cover the scaffolds within 21 days in a homogeneous layer, and the boxes become almost undistinguishable (**Figure** **6**a), and also myotube‐like structures can be observed.

To obtain a more detailed view of the differentiation, gene expression analysis of Myod1 and myogenin was executed. C2C12 cells cultured in differentiation media exhibited higher Myod1 expression than in growth media, except for spheroids cultivated on tissue culture plate (TCPs) on days 14 and 21. Overall, Myod1 expression in C2C12 single cells was higher on fibrils than on TCP, except on day 1. Spheroids showed higher Myod1 expression on fibrils on days 5 and 21 and days 14 and 21 in differentiation media (Figure 6b).

## 3 Discussion

This study aims to show the potential of collagen‐mimicking nanostructures derived from meltelectrofibrillation as tools for supporting maturation and myogenesis of C2C12 cells. The material here proposed adds to the efforts of bioengineering synthetic matrices for tissues by mimicking the ECM and offering cells support, guidance, and environmental cues for maturation,^[^ ^19^ ^]^ overcoming the limitations of traditional platforms relying on 2D fiber substrates.

The scaffolds are printed by Melt electrowriting to produce highly organized fibers (with a diameter of 10 µm) of a PCL‐PVAc blend. The melting of the polymer blend of immiscible polymers causes a phase separation in the melt, and once pressure is applied for extrusion, the material flows through the nozzle, where shear forces cause the alignment of the polymer phases. In this case, PCL disperses in a matrix of PVAc, and upon meltelectrowriting, the polymer chains stretch and elongate to form fibrils. Printing the fibers allows to organize the overall scaffold geometry precisely and reproducibly.

After printing the fibers and washing the PVAc away with ethanol, nano‐to‐micro scale PCL fibrils organised as bundles remain. These microstructures resemble the natural structure of collagen, and are therefore very interesting for studying collagen‐rich environments.^[^ ^20^ ^]^ Thanks to the precision printing of the fibers it is possible to recapitulate highly aligned fibrous matrices.^[^ ^21^ ^]^ The scope of this work is to examine the effect of such synthetic matrix on cells. In particular, since the muscle shows this hierarchical structure, we are interested in their effect on muscle cells organized in spheroids.

Spheroids are excellently suited as tissue building blocks since the cells partially mature for a few days during the spheroid formation and cultivation and are ultimately seeded on the scaffold at a later time. Advantages of this are in the early assembly of the cells in a 3D micro‐tissue that can already convey important aspects of cell‐cell interaction and ECM deposition.^[^ ^22^ ^]^ The spheroids in this study have been produced by seeding microwells produced with the use of negative micropillar templates out of PDMS for agarose casting (Figure 2). This method provides a self‐sufficient way of easily producing a high number of spheroids without extensive effort.^[^ ^23^ ^]^ Differently sized spheroids were formed proportionally to the number of cells per well, showing good size distribution and also good viability over time. Better results are achieved with smaller spheroids, however, most of the dead cells in the larger ones could be due to handling since they are present on the outer edges.

The fibrillar scaffold was designed to contain the spheroids, therefore the shape of a box was chosen (Figure 1).^[^ ^24^ ^]^ The boxes have been filled with spheroids for the seeding of a high number of cells and different amounts of spheroids per box were tried to optimize the volume to number of cell ratio. After 5 days of cultivation it was observed how spheroids tend to disassemble upon interaction with the scaffold and the cells favor the migration along the fibrils. Interestingly, from SEM imaging of the scaffold cross‐section through Focused Ion Beam we can observe cell infiltration within the bundles. Therefore, the cells organization is strongly affected by the fibrillar structures, not only taking advantage of the highly aligned surface cues for distributing spatially, but also colonizing the structure three‐dimensionally within 5 days. This finding is relevant for muscle tissue engineering applications since it demonstrates how these fibrils guide cells into the hierarchical strucures needed when recapitulating the muscular tissue.^[^ ^25^ ^]^ Moreover, the PCL fibrils offer mechanical stability for handleability of the constructs during maturation.

Spheroids offer high cell density and promote enhanced cell–cell contact prior to scaffold seeding, simulating early developmental stages of muscle formation. This configuration facilitates rapid fusion and alignment of cells along the anisotropic fiber topography. These features contributed to the early formation of myotube‐like structures, as confirmed by confocal microscopy and further supported by the increased expression of maturation markers. To enhance cell's maturity, in conjunction with the fibrils, differentiation media has been employed.^[^ ^26^ ^]^ Despite the high number of cells per well even a longer culture time showcases an excellent viability in both cases with a better outcome for the differentiation media. As also shown from the DNA content amount.

So far only a visual description of cell behaviour has been provided, the proof that this material is suitable for muscle cell maturation is shown with the production of myosin by the cells that not only align along fibril direction but also forms longitudinal cells bundles amenable to early‐stages myotubes, with a better outcome for the cells cultured in differentiation media. Prolonged culture of spheroids on electrofibrillated scaffolds under differentiation conditions led to sustained expression of key myogenic markers, including Myod1 and Myogenin, in comparison to 2D control cultures (Figures 5 and 6; Figure S4, Supporting Information). This suggests enhanced and prolonged myogenic commitment supported by the synergistic effects of nanofibrillar topography and 3D spheroid organizations.^[^ ^27^ ^]^ The simultaneous upregulation of both early (MyoD) and intermediate (Myogenin) myogenic transcription factors in spheroid‐seeded constructs points to a modulation of the canonical myogenic program. This is likely facilitated by the 3D microenvironment provided by the spheroids, which promotes enhanced paracrine signaling and direct cell–cell interactions.^[^ ^28^ ^]^ These features may contribute to a more synchronized and sustained activation of the myogenic cascade, emphasizing the advantage of the spheroid‐based approach for achieving uniform and robust myogenic differentiation. Importantly, immunostaining with the MF20 antibody, specific for myosin heavy chain II, a hallmark of mature myotubes, confirmed late‐stage differentiation. Strong MF20 signals were particularly prominent in spheroid‐seeded scaffolds and were accompanied by the formation of elongated, aligned myotube‐like structures. Quantitative analysis of myotube length further supported the structural and functional maturation of the engineered muscle tissue. Interestingly, cells cultured on electrofibrillated scaffolds, whether as spheroids or as single cells, consistently outperformed 2D controls under both growth medium (GM) and differentiation medium (DM) conditions. This underscores the role of nanofibrillar scaffold topology in providing topographical cues that promote myogenic differentiation. Notably, spheroids on fibrillated scaffolds demonstrated the highest degree of myogenic commitment and myotube maturation, even under GM conditions, reaching levels comparable to single‐cell cultures in DM. This finding suggests that the topography of the nanofibrillar scaffold, combined with enhanced cell–cell contact within spheroids, may contribute to improved paracrine signaling and support myogenic differentiation, even under conditions with limited differentiation factors in the media.

Taken together, these results demonstrate that the combination of electrofibrillated scaffold architecture and spheroid‐based bioassembly not only enhances early myogenic commitment but also supports sustained progression toward mature myofiber formation. The use of C2C12 cells provides a valuable proof‐of‐concept for the myoinductive potential of the collagen‐mimicking scaffold. For translational applications, future studies should quantitatively assess differentiation efficiency and fusion index, incorporate human‐derived cell sources such as iPSC‐derived muscle progenitors, and apply advanced modeling strategies. These may include co‐culture with endothelial or neuronal cells, mechanical stimulation (e.g., stretching), and dynamic bioreactor‐based culture. Such refinements would enable the development of a more physiologically relevant, organoid‐like platform for studying human muscle development, disease modeling, and therapeutic screening.

## 4 Conclusion

The effect of the ECM on cells is notably important in tissue engineering and in the vast majority of tissues, highly hierarchical structures, such as alignment of the matrix, components is fundamental. While natural collagen is an expensive and a not easy to work with material, especially to recreate anisotropic structures, we aim to provide a solid platform that mimics the super structure of collagen for the development of anisotropic tissues and facilitation of cell hierarchical organization. A synthetic matrix offers stability, reproducibility, and ease of handling, moreover these matrices can be coated to offer the biochemical cues to cells. In this study we showed that microfibrillar structures deriving from the electrofibrillation of PCL, are a powerful tool for providing environmental cues to cells, muscle cell spheroids in particular. Meltelectrofibrillation guides cell's behavior not only of the single cell but also of cell aggregates, such as spheroids, which disaggregate in an organized manner following along the fibers. After more cultivation time the aligned cells further organize to form myotubes and express myogenic transcription factors. Conclusively, we demonstrated the potential of meltelectrofibrillation for the biofabrication of anisotropic tissues such as muscles. proving our method suitable for such application and as a valid alternative to the current available techniques.

## 5 Experimental Section

### Melt Electrofibrillation

The nanofibrous scaffolds were fabricated using melt electrowriting of a polymer blend. Three different compositions were printed: 70%–30%, 75%–25%, and 80%–20% of polyvinyl acetate (PVAc) and polycaprolactone (PCL), respectively. The polymer blend was melted at 175 °C and extruded through a heated nozzle (160 °C) under 1 bar pressure. A 3 kV voltage was applied during printing, with a nozzle‐to‐collector distance of 3.3 mm. The scaffolds were printed with two different geometries: squared (0.5 mm × 0.5 mm) and rectangular (1 mm × 0.5 mm) box structures. Catching fibers were incorporated at the bottom of the boxes to facilitate the capture of spheroids.

To induce fibrillation, PVAc was selectively removed by washing the printed scaffolds in 70% ethanol three times, with each incubation lasting 1 h.

Scaffolds were imaged through scanning electron microscopy (SEM) (Zeiss Crossbeam 340 Field‐Emission Electron Microscope, Zeiss Microscopy, Oberkochen, Germany) and were previously sputter‐coated with a 4 nm thick platinum layer (Leica EM ACE600, Leica Microsystems GmbH, Wetzlar, Germany).

### Cell Culture and Spheroid Formation

Murine myoblasts C2C12 (ATCC CRL‐1772, Manassas, VA, USA) cells were cultured in Dulbecco's Modified Eagle medium (DMEM) (41965‐039, Gibco, Thermo Fischer Scientific, Waltham, USA) media provided with 10% inactivated FCS, 100 U mL^−1^ Penicillin, and 0.1 mg mL^−1^ streptomycin. Spheroids were fabricated using agarose hydrogels in the shape of honeycomb‐inspired cell‐clustering microwells. To create these microwells, master molds were first 3D printed using a Prusa SL1S DLP printer (Prusa Research, Prague, Czech Republic)) in FotoDent resin (Dentamid Fotodent 405 nm, Dreve, Dresden, Germany). A polydimethylsiloxane (PDMS) (Sylgard 184 Silicone Elastomer, Dow, Midland, MI, USA) stamp was then fabricated by casting PDMS into the 3D‐printed microwells to form micropillars. Once cured, the PDMS stamp was used to replicate the honeycomb‐inspired microwell pattern in agarose. For the fabrication of the microwells, a 2% agarose solution was prepared by dissolving agarose (Sigma–Aldrich, Darmstadt, Germany) in distilled water and heating it in a microwave oven. The PDMS‐based micropillar molds were placed in 35 mm diameter Petri dishes and covered with the molten agarose solution. After hydrogel formation, the microwells were sterilized by incubation in 70% ethanol for 30 min, followed by six washes with sterile PBS, with 10 min incubation in every alternate wash. The hydrogels were then kept overnight in PBS, exposed to 254 nm UV light for 30 min the following day, and incubated in culture media for 10 min before cell seeding. The microwell‐containing hydrogels were transferred to a 24‐well plate and covered with 500 µL of cell suspension in growth media. C2C12 cells were seeded at concentrations of 0.5 × 10⁵, 1 × 10⁵, 2 × 10⁵, 5 × 10⁵, and 10 × 10⁵ cells per hydrogel. The samples were then centrifuged at 1,000 rpm for 3 min and incubated at 37 °C for up to 24 h to allow spheroid formation. The spheroids were imaged with an optical Zeiss microscope, and the images were analyzed using Image J.

### Viability

The viability of cells on scaffolds was evaluated using the Live and Dead staining kit (Thermo Fischer Scientific, Waltham, USA) on the 5th and 21st day of culture. Briefly, cells were treated with a working solution of Calcein AM (1:1000) and ethidium‐homodimer (1:500) in DPBS for 30 min under standard culture conditions. After that, images were captured using a confocal microscope (Leica LSM SP8, Germany).

### Scaffold Seeding

The PCL scaffolds were sterilized with 70% ethanol under UV for 30 min and washed with PBS five times. The washed scaffolds were then fitted into custom‐designed, 3D‐printed inserts consisting of two parts: a bottom section (holding frame) that held the scaffold and a top component (retaining insert) that secured it in place. The inserts were designed using Fusion 360 (Autodesk Inc., San Rafael, CA, USA) and fabricated with a Prusa SL1S Digital Light Processing (DLP) printer in FotoDent resin. Inserts were closed with a glass slide on the bottom and sterilized under UV light for 1 h. After transfer to a 6‐well plate, they were incubated in prewarmed media. This study added the 680 spheroids per scaffold, the spheroids were collected and resuspended, afterward they were pipetted on the top of the scaffolds; spheroids distribute homogeniously in the boxes according to spheroid suspention concentration. Cells were maintained in growth media conditions for five days.

On cultivations longer than day 5, myogenic differentiation media (DM) was used for the C2C12 cells. The DM was composed of DMEM (41965‐039, Gibco, Thermo Fischer Scientific, Waltham, USA) supplemented with 2% horse serum (Thermo Fischer Scientific, Waltham, USA), and 1% pen/strep.

Scanning electron microscopy images of the scaffolds were performed after sample prepping: scaffolds were washed four times with PBS before fixation with 6% Glutardialdehyde on ice for 15 min. Afterward, scaffolds were washed with PBS on ice for 10 min and, for dehydration, subjected to an ethanol series. Incubation for 15 min with hexamethyldisilazane (HMDS) twice and evaporation overnight ensured a thoroughly dried scaffold. Then, scaffolds were mounted and sputtered with a 4 nm platinum layer using a sputter coater (Leica EM ACE600, Leica Microsystems GmbH, Wetzlar, Germany). Electron microscope images with a focus ion beam‐SEM (FIB‐SEM) were taken with a Zeiss Crossbeam 340 Field‐Emission Electron Microscope (Zeiss Microscopy, Oberkochen, Germany) at an acceleration voltage of 2 kV.

### Immunostaining

An F‐actin staining was performed to analyze the cell morphology and alignment. Briefly, scaffolds were washed with PBS and fixed in 4.5% formalin at room temperature for 1 h. Then, samples were washed thrice in PBS and incubated in a working solution of Phalloidin Rhodamine 555 (1:1000) (Abcam, Cambridge, UK) that was applied at room temperature in the dark for 1 h. After washing in PBS, the scaffolds were mounted with DAPI mounting medium (Dianova, Hamburg, Germany) and analyzed using a confocal microscope. The cell alignment was evaluated using ImageJ and Excel.

### Myosin Staining

The samples were fixed and blocked, as described earlier. Afterward, the fixed scaffolds were washed briefly with PBS before 0.1% Triton was added for 10 min. Scaffolds were blocked with 2% bovine serum albumin (BSA) in PBS at room temperature for 1 hr. Then the scaffolds were incubated with Anti‐Myosin 4 eBioscience (1:200) (Invitrogen, Thermo Fischer Scientific, Waltham, USA) at 4 °C overnight. Following the scaffolds were washed with PBS and incubated with the secondary antibody (Alexa Fluor 488 AffiniPure Goat Anti‐Mouse IgG + IgM, Jackson ImmunoResearch Europe Ltd, Cambridgeshire, United Kingdom) in 1% BSA in PBS at room temperature in the dark for 3 h. The scaffolds were mounted with DAPI mounting medium after washing twice shortly and once for 5 min with PBS. Images were taken with the confocal microscope. Myotube measurements were conducted with ImageJ and Microsoft Excel.

### Myogenic Gene Expression

We collected samples (n = 3) on 1, 5, 14, and 21 days of culture on the fibrillated scaffold. Control samples consisted of the same number of spheroids seeded onto standard 2D well plates under identical media conditions.

The samples were homogenized in Trizol (Thermo Fischer Scientific, Waltham, USA) and stored at −80 °C until further use. Chloroform was added to the thawed samples and after a 7 min incubation and 5 min centrifugation at 12 000 g, phase separation was achieved and the clear upper phase containing the RNA was transferred to a new reaction tube. This solution was incubated for 10 min with isopropanol on ice to precipitate the RNA. After centrifugation at 12,000 g for 10 min at 4 °C, the RNA was washed twice with 75% Ethanol. The RNA pellet was dried and afterward dissolved in diethyl pyrocarbonate (DEPC)‐H2O. The RNA concentration and purity were measured using a TECAN Spark 20 M plate reader, and the RNA was stored at −80 °C until further use.

The complementary DNA (cDNA) was synthesized from 500 ng RNA using the high‐capacity cDNA reverse transcription kit, which was carried out according to the manufacturer´s instructions. The cDNA reaction was performed with the MiniAmp Thermal Cycler (Thermo Fisher Scientific, Waltham, USA) with the temperature course of 25 °C for 10 min, 37 °C for 120 min, 85 °C for 5 min, and a 4 °C hold. Afterward, the cDNA samples were diluted with DEPC‐ H2O to an end concentration of 5 ng µL^−1^ and stored at −20 °C.

The myogenesis on the scaffolds was further evaluated on gene expression level via qPCR using the StepOnePlus real‐time PCR system with SYBR select master mix. About 5 ng of cDNA were analyzed for the transcription factors myogenin, MyoD1 (For: TGCTCTGATGGCATGATGGA, 100 nm and Rev: GTAGTAGGCGGTGTCGTAGC, 200 nm), and Myog (For: GTCCCAACCCAGGAGATCATTT, 100 nm and Rev: GTCCACGATGGACGTAAGGG, 100 nm), which were characteristic myogenic markers. The murine eukaryotic elongation factor 1 (mEef1, For: AGTGTCCTGAGCAAGTGGTG, 200 nm and Rev: AGATCAGCGGTGAAGCCAAA, 200 nm) was employed as a housekeeping gene. The qPCr was performed with the following temperature run: 50 °C for 2 min, 95 °C for 2 min, 40 cycles of 95 °C for 15 s and 60 °C for 1 min, followed by 95 °C for 15 s, 60 °C for 1 min, increase in 0.3° steps to 95 °C in 15 s intervals. Threshold cycle (Ct) values were analyzed with the StepOne Software, and the further analysis was done with Microsoft Excel.

### Proliferation of C2C12 Using DNA Assay

The scaffolds were washed with PBS and then lysed with 0.5% Thesit. In order to ensure cell lysis throughout the scaffold, samples were treated with ultrasound (0.2 s intervals for 2 s at a 10% amplitude). Samples were pipetted in duplicates of 100 µL in a black 96‐well plate. 100 µL duplicates of a deoxyribonucleic acid (DNA) ladder were added to the well plate. 100 µL of PicoGreen reagent (1:200 diluted in 1xTE buffer) was added to all wells. After a 2 min incubation, the fluorescence intensity of the samples was measured with the TECAN with 485 nm excitation and 538 nm reference wavelength.

### Statistical Analysis

All quantification data values were presented as the means ± standard deviations (*n* = 4).  The statistical significance was evaluated using Student's t‐test and one‐way ANOVA (one variable), while two‐way ANOVA (two variables) was performed for the DNA assays using Origin software. A p‐value less than 0.05 was considered significant.

## Conflict of Interest

The authors declare no conflict of interest.

## Supporting information



Supporting Information

## Data Availability

The data that support the findings of this study are available from the corresponding author upon reasonable request.
