# Supplementary material for: Bioassembly of Myoblast Spheroids in Electrofibrillated Scaffolds for 3D Muscle Tissue Biofabrication
Source: Small. 2025 Aug 28;21(41):e03351. doi: 10.1002/smll.202503351 (PMC12530036; doi:10.1002/smll.202503351)
Supplement: Supplementary file 1 — Supporting Information [file SMLL-21-e03351-s001.docx]

Supporting Information

**Bioassembly of Myoblast Spheroids in Electrofibrillated Scaffolds for 3D Muscle Tissue Biofabrication**

Co

rinna Heinze^1+^, Camilla Mussoni^1+^, Matthias Ryma^1,2^, Csaba Gergely^1^, Zan Lamberger^1^, Anna Schäfer^1^, Kristina Andelovic^1^, Philipp Stahlhut^1^, Gregor Lang^1^, Jürgen Groll^1^*, Taufiq Ahmad^1^*

C. Heinze, C. Mussoni, M. Ryma, C. Gergely, Z. Lamberger, A. Schäfer, K. Andelovic, P. Stahlhut, G. Lang, J. Groll, T. Ahmad

Department of Functional Materials in Medicine and Dentistry, Institute of Functional Materials and Biofabrication (IFB), and Bavarian Polymer Institute (BPI), Julius-Maximilians-Universität Würzburg, 97070 Würzburg, Germany.

M. Ryma

Chair of Biomaterials, Engineering Faculty, University of Bayreuth, Prof.-Rüdiger-Bormann-Straße 1, 95447 Bayreuth, Germany

* Authors to whom any correspondence should be addressed
E-mail: [juergen.groll@uni-wuerzburg.de](mailto:juergen.groll@uni-wuerzburg.de), [taufiq.ahmad@uni-wuerzburg.de](mailto:taufiq.ahmad@uni-wuerzburg.de)


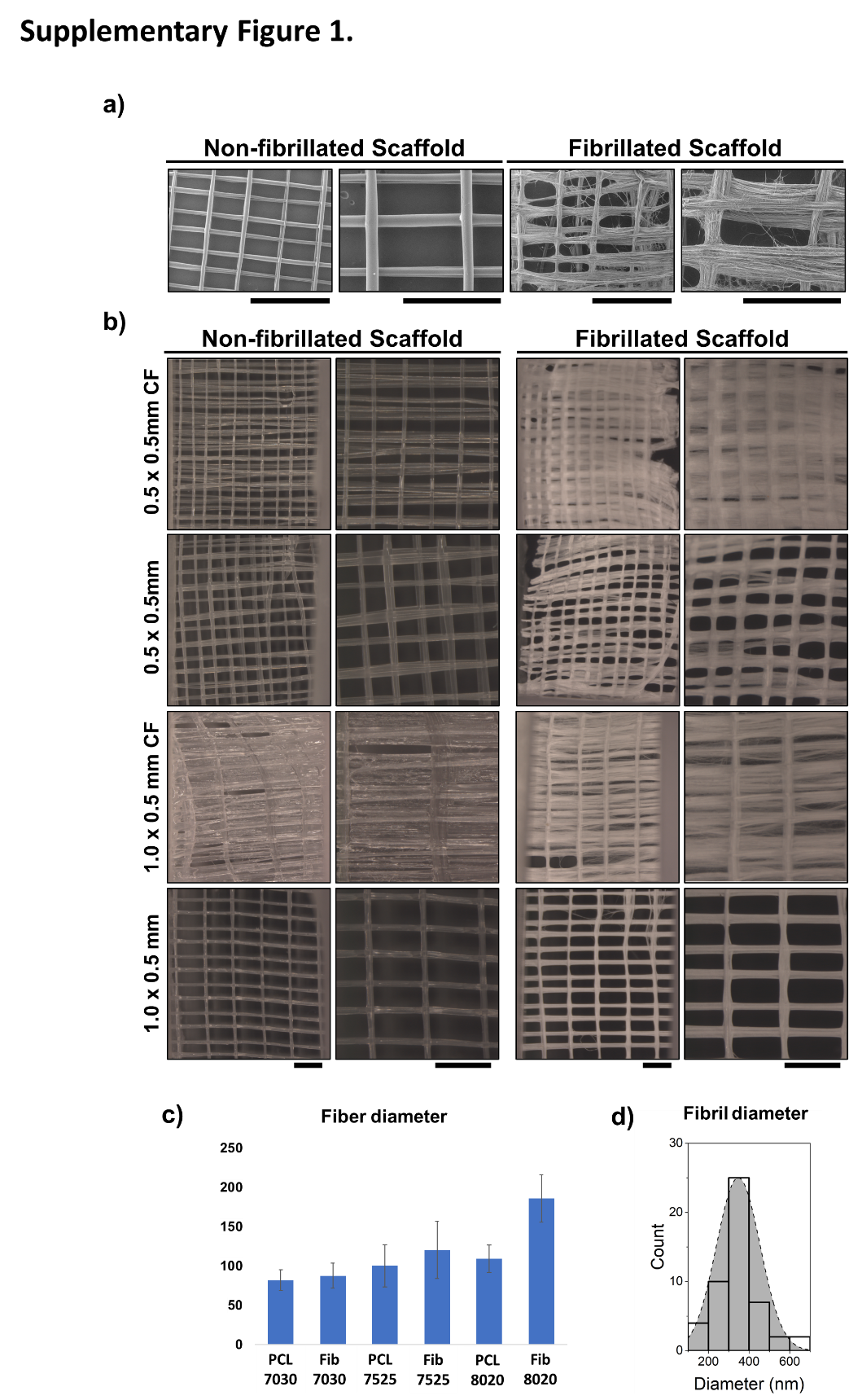
Supporting Information

**Figure S1.** A) Scanning Electron Microscopy (SEM) of non-fibrillated scaffolds and fibrillated scaffolds at different magnifications. Scale Bar: 2 mm (left), 1 mm (right). B) Optical microscope imaging of the non-fibrillated scaffolds and relative fibrillated scaffolds in a squares geometry and rectangular geometry, with and without bottom catching fibers (CF). Scale Bar: 1mm. C) Fibril diameter distribution.


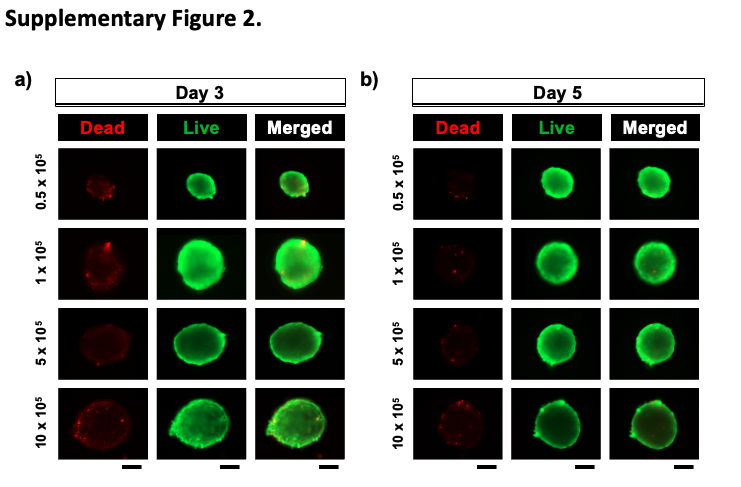


**Figure S2.** A) Live dead of the C2C12 spheroids of different size and cell number on day 3 of culture. B) Live dead of the C2C12 spheroids of different size and cell number on day 5 of culture.


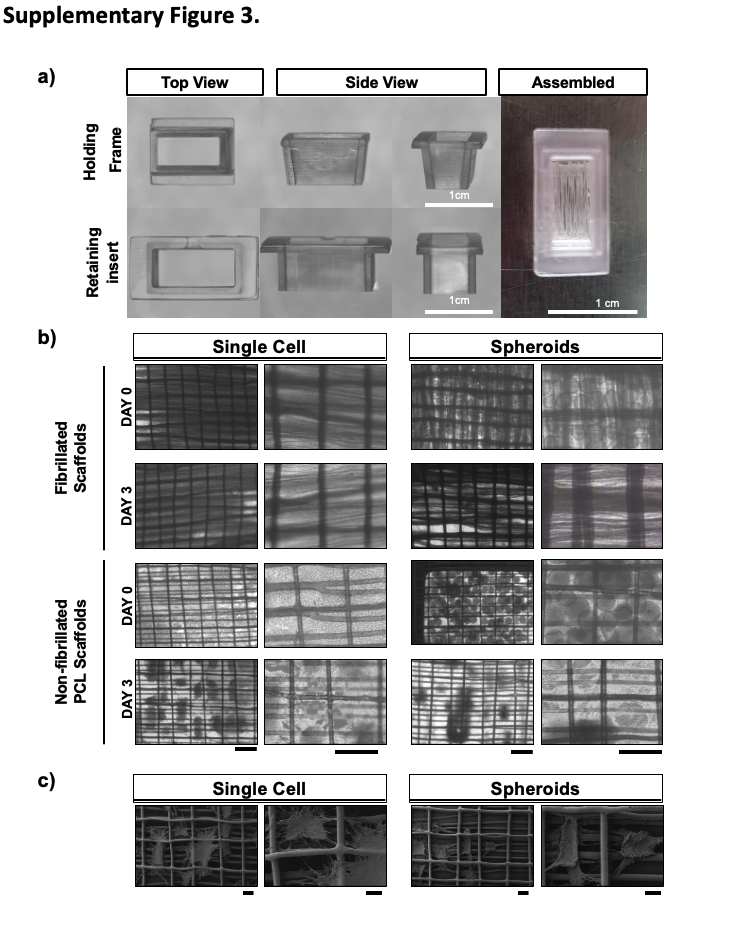


**Figure S3.** A) 3D printed holders for supporting the scaffolds for spheroid seeding. B) Optical microscope imaging of single cell and spheroid seeded scaffolds over 3 days a, fibrillated and non-fibrillated, at different magnification. Scale Bar: 1mm. C) SEM imaging of the non-fibrillated scaffolds seeded with single cells (left) and spheroids (right). Scale Bar: 1mm (left), 0.2 mm (right).


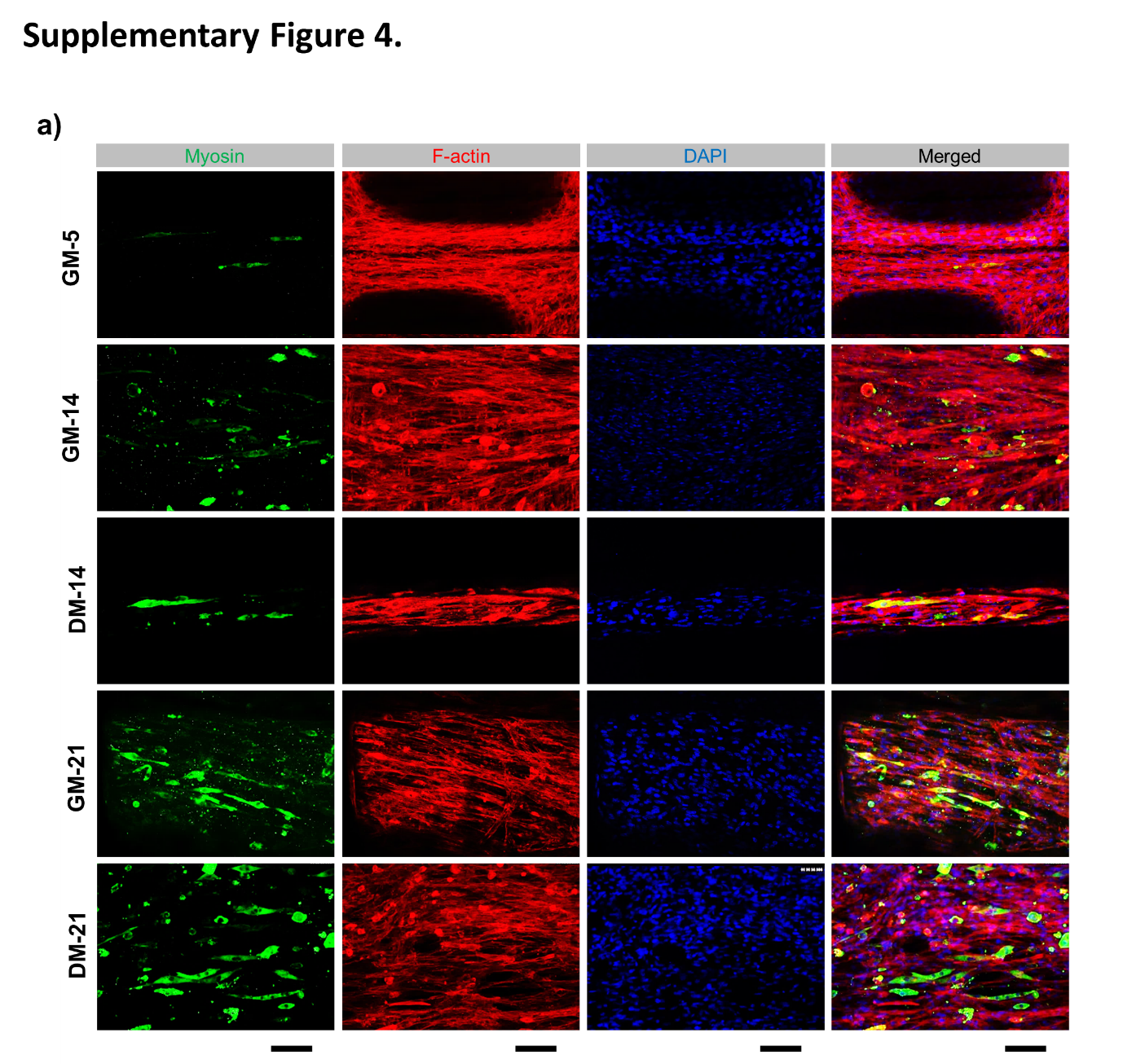


**Figure S4.** Myosin, f-actin and dapi staining of the scaffolds on day 5, 14 and 21 for the scaffolds cultured with growth media (GM) or differentiation media (DM). Scale Bar: 1 mm.
